# Capped Plasmonic Gold and Silver Nanoparticles with Porphyrins for Potential Use as Anticancer Agents—A Review

**DOI:** 10.3390/pharmaceutics16101268

**Published:** 2024-09-28

**Authors:** Nthabeleng Hlapisi, Sandile P. Songca, Peter A. Ajibade

**Affiliations:** School of Chemistry and Physics, University of KwaZulu-Natal, Private Bag X01, Scottsville, Pietermaritzburg 3209, South Africa; 221121728@stu.ukzn.ac.za (N.H.); songcas@ukzn.ac.za (S.P.S.)

**Keywords:** silver, gold, nanoparticles, cancer, photothermal therapy, porphyrins, photodynamic therapy

## Abstract

Photothermal therapy (PTT) and photodynamic therapy (PDT) are potential cancer treatment methods that are minimally invasive with high specificity for malignant cells. Emerging research has concentrated on the application of metal nanoparticles encapsulated in porphyrin and their derivatives to improve the efficacy of these treatments. Gold and silver nanoparticles have distinct optical properties and biocompatibility, which makes them efficient materials for PDT and PTT. Conjugation of these nanoparticles with porphyrin derivatives increases their light absorption and singlet oxygen generation that create a synergistic effect that increases phototoxicity against cancer cells. Porphyrin encapsulation with gold or silver nanoparticles improves their solubility, stability, and targeted tumor delivery. This paper provides comprehensive review on the design, functionalization, and uses of plasmonic silver and gold nanoparticles in biomedicine and how they can be conjugated with porphyrins for synergistic therapeutic effects. Furthermore, it investigates this dual-modal therapy’s potential advantages and disadvantages and offers perspectives for future prospects. The possibility of developing gold, silver, and porphyrin nanotechnology-enabled biomedicine for combination therapy is also examined.

## 1. Introduction

Noble metals like gold (Au), silver (Ag), platinum (Pt), and copper (Cu) hold a historical significance. Still, their primary use in ancient cultures is significantly different from their modern applications due to the development of nano-based products from them [1,2,3]. These metals are being used extensively in electronics, medicine, and catalysis. When structural materials are fabricated into nanostructured materials, their size-dependent properties open up a whole new range of light-related applications, such as the development of novel therapeutics agents [4]. Attention in turning the interactions between light and metals to create nanoplasmonics has led to novel applications beyond merely reflecting light [5,6]. Ag and Au nanoparticles, among other metal nanoparticles (MNPs), have the most intriguing physicochemical characteristics for bio-applications [7]. AgNPs offer enhanced results in terms of sensitivity in biological application, while AuNPs continue to be the most researched in the field due to their notable chemical stability and biocompatibility. Localized surface plasmon resonance (LSPR), which gives colloidal nanoparticles their vibrant color, is one of the key distinctive physical characteristics of metallic NPs [8]. Since AuNPs and AgNPs have strong interactions with light, these materials are specifically studied for their optical properties [9,10]. 

The ancient Greek and Roman Empires were the pioneers in the application of Ag as an antibiotic and antimicrobial agent [11]. At that time, silver’s therapeutic and preservative properties were mostly utilized to make water and other liquids consumable and protect vessels from bacterial infections. They were already renowned as a powerful tool against the development of pathogens. The interaction of silver ions with the thiol groups of essential bacterial enzymes and proteins, which results in cell death, gives silver its antibacterial properties [12,13,14]. 

Undeterred by scientific attempts, cancer is one of the world’s most formidable health challenges, defined by the aberrant growth and spread of malignant cells [15]. This is because cancer cells are very diverse and complex; hence, it is difficult to find a congruent therapy [16,17]. One of the most common cancers in Africa is skin cancer, where reports have shown that in South Africa alone, about 20,000 cases are reported annually, with around 700 deaths. On the other hand, the World Health Organization indicates that 2–3 million skin cancer melanomas and 137,000 melanomas are reported annually around the globe [18]. In 2020, approximately 18.1 million newly identified cases of cancer (except non-melanoma skin cancer) were diagnosed worldwide, with lung cancer accounting for the majority (12.4%) [19]. Other common malignancies include breast, colon, rectal, and prostate. The lifetime risk of developing cancer is exceptionally high, with about one in every five people predicted to develop the disease; one in every eight men and one in every eleven women die from it [20]. Future forecasts are equally troubling, with a 31% increase in early-onset cancer incidence and a 21% increase in associated fatalities by 2030 [21]. 

**Figure 1:**
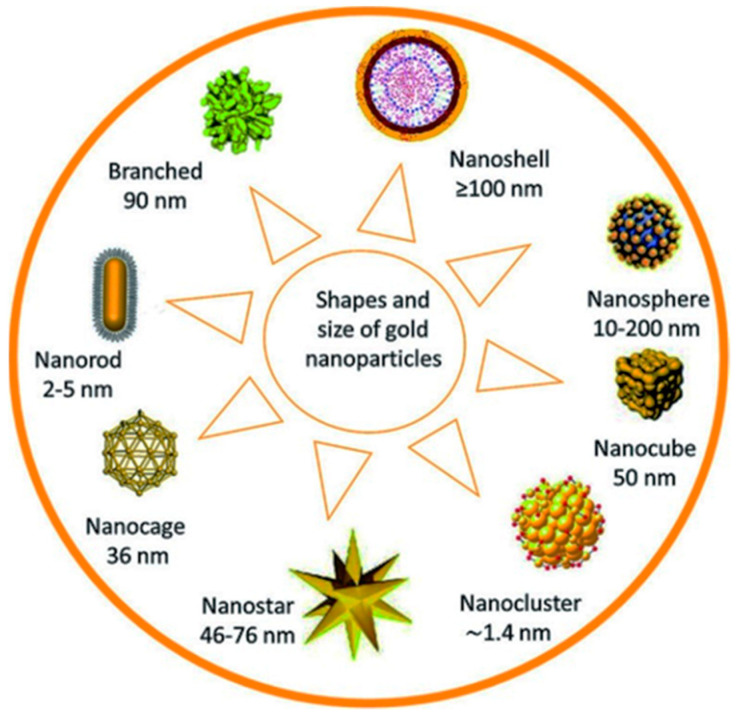
Different gold nanoparticles and their sizes.

Over the past decade, nanotechnology has been explored to develop novel therapeutic and anticancer agents [22]. This is due to the peculiar properties that nanomaterials have shown over traditional treatment methods. Nanomaterials, among other uses in nanomedicine, have been used in drug delivery and specific targeting [23]. Besides these, nanomaterials are relatively better than conventional treatment methods because they enhance the therapeutic effect, reduce toxicity and have higher biocompatibility [24]. The inimitable properties of nanoparticles (NPs), for example, their size, shape, and large surface area, enhance their potential in treating diseases. This potential of nanoparticles in therapy has been extensively explored, and findings show that the MNPs can be used in imaging [25], drug delivery [26], tissue engineering [27], biosensing, and cancer therapy [28]. Nanotechnology has provided a different platform for the consignment of therapeutic nano-drugs in the body, providing relatively better specificity and retention [29,30]. Specifically, AuNPs have high tumor retention properties owing to their natural affinity to leaky tumor growth. Figure 1 below shows different shapes of gold nanoparticles utilized for bio-application [31,32].

While the first colloidal gold syntheses predate much of the peer-reviewed literature, Michael Faraday discovered in 1857 that the “fine particles” developed by the aqueous reduction of gold chloride by phosphorus, which might be stabilized by the addition of carbon disulfide, leading to a “beautiful ruby fluid.” [33]. Most colloidal synthetic methods used for the preparation of gold nanoparticles to date use a similar strategy, in which a solvated gold salt is reduced when surface capping ligands are present, which prevents the particles from aggregation due to electrostatic and physical repulsion [34,35]. The ratio of gold ion/reducing agent or gold ion/stabilizer influences particle size and monodispersity of the as-prepared gold nanoparticles [36,37].

Properties of AuNPs, such as their surface plasmon resonance (SPR) and their capacity to bind on thiol and amine groups, allow for alteration and application in biomedicines [38,39]. Although the biological mechanism of gold nanoparticles has been debated for a long time, nonspecific receptor-mediated endocytosis is the most likely path [40]. Experimental data from in vivo studies showed that gold nanoparticles with or without functionalization tend to accumulate in tumor cells. The effect is enhanced permeability and retention (EPR) [41,42]. At times, however, the improved retention and permeability phenomena cannot be utilized, which occurs when the tumor is heterogeneous. The particle uptake and reticuloendothelial system (RES) occurs [43]. One of the systems to counter RES is PEGylation; this entails the preparation of a hydrated barrier, which will, in turn, cause steric hindrance to the attachment of phagocytes [44]. AuNPs are good photothermal therapy (PTT) agents because they convert light to thermal energy to kill malignant cells [45,46]. 

AgNPs are among the metallic nanoparticles that have been explored potentially for treating antimicrobial-resistant infections and cancer [47]. They have been synthesized using biological and chemical methodologies. However, the green synthesis method is preferred over the chemical method because it is more environmentally friendly, as it uses plant extracts. Silver nanoparticles are synthesized using different methods to yield size- and shape-controlled nanoparticles used for different purposes [48]. The tuning of nanoparticles for specific purposes has interested researchers in the past years [49,50]. An example is when silver nanorods are capped and used for their potential as anticancer agents, their antioxidant effects, and their accumulation and retention in tissues [51,52].

**Figure 2:**
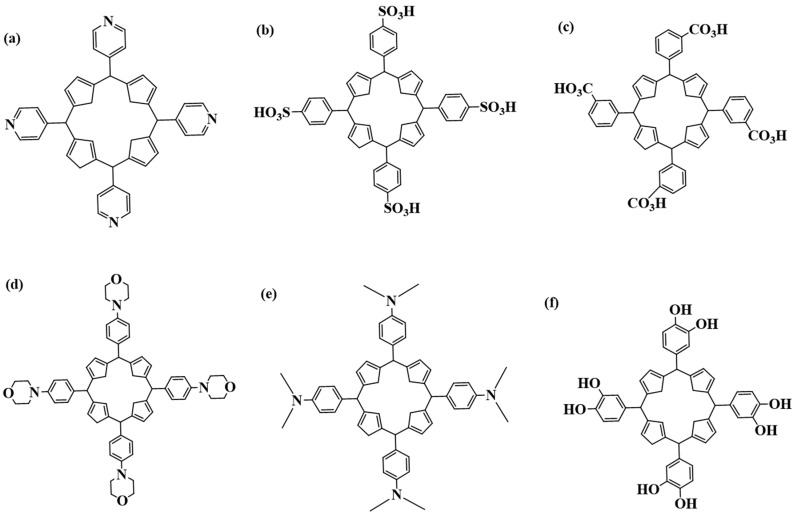
Porphyrins used for the potential treatment of cancer: (**a**) 5,10,15,20 tetrakis(pyridyl)porphyrin; (**b**) meso-tetrakis-(4-sulfonatophenyl)porphyrin; (**c**) tetra-3-carboxyphenyl porphyrin; (**d**) meso-tetrakis-(morpholine)porphyrin; (**e**) 5,10,15,20 tetrakis(dimethylaniline)porphyrin; (**f**) Tetrakis(benzene-1,2-diol)porphyrin.

Generally, metal nanoparticles (MNPs) have a broad spectrum in treating cancer; however, to improve targeting, the nanoparticles can also be encapsulated with organic molecules like porphyrins. Porphyrins are a group of macrocyclic organic molecules that have been thoroughly researched for their ability to treat anti-resistant bacteria and cancers [53,54]. Figure 2 below shows some cationic porphyrins that have been used for the potential treatment of different cancers. Cationic porphyrins are utilized in PDT owing to their strong ability to generate singlet oxygen upon light activation, which is required for causing cell death in targeted cancer cells. Their positive charge promotes cellular absorption and interaction with negatively charged cell membranes, increasing therapy efficacy. In addition, their adaptable chemical structure enables simple modifications to improve photophysical and biological properties.

**Figure 3:**
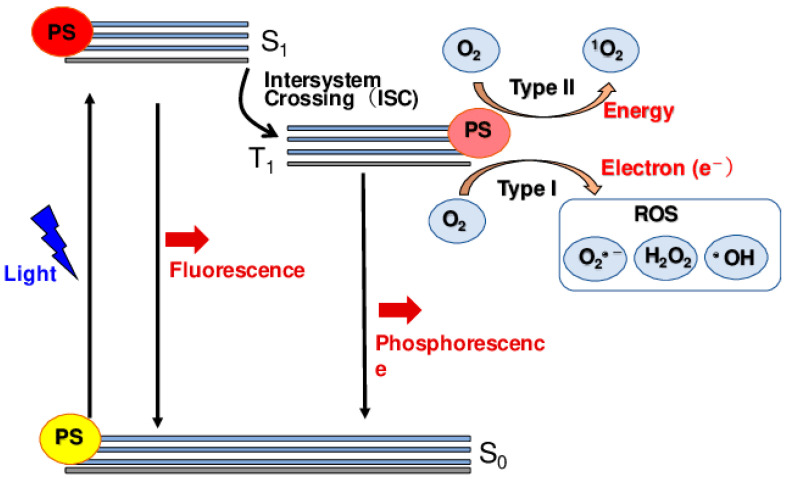
Singlet oxygen production by the photosensitizing process [62].

Ongoing research on the use of porphyrins in photodynamic therapy (PDT) has drawn the attention of many scientists due to their favorable physicochemical properties [55]. The principle behind PDT is that the photosensitive molecule accumulates mainly on the tumor cells but not on the non-malignant cells [56]. The cells are then irradiated with light of a definitive wavelength to kill the cell (tumor) [57]. The excitation of the photosensitizer in the company of molecular oxygen will, in turn, produce singlet oxygen, 1O2 (photodynamically active agent), as seen in Figure 3. Ideally, only the irradiated cells will be destroyed, and hence, PDT is gaining popularity. Porphyrins are unique molecules that find favor in their extended π system, making them have a high extinction coefficient, leading them to be highly hydrophobic [58,59]. Additionally, meso-functionalization of these molecules results in them being extremely water-soluble. PDT predominantly uses the type II mechanism where porphyrins or their counterparts are used to be photosensitizers [60,61]. Since PDT is dependent on oxygen, in cases of a phenomenon where tumor cells grow even in low oxygen supply, the efficacy of PDT is reduced when the partial oxygen pressure pO2 is below 40 mmHg [62,63,64]

**Figure 4:**
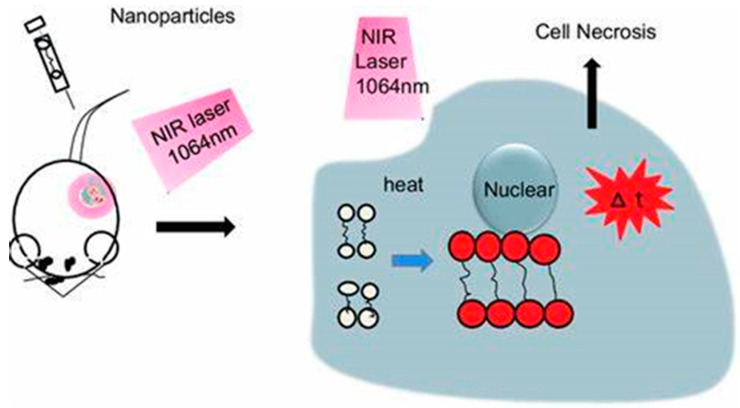
Mechanism of PTT.

In cases of hypoxia, other modalities should be used in conjunction with PDT, such as PTT, which is not oxygen-dependent. On the other hand, PTT is light-dependent; irradiation of light of a distinct wavelength causes the PTT agent to absorb the light and disperse the light through non-radiative decay [65,66]. Temperatures will gradually rise in the localized environment, leading to irreversible cell damage, as Figure 4 shows. Gold nanoparticles are excellent PTT agents because they possess a surface plasmon resonance (SPR) oscillation, and their high absorption crosses the near-infrared region (NIR) [67,68]. There have been different studies on the functionalization of AuNPs and AgNPs, and this review explores some of these studies, with emphasis on those with enhanced photothermal and photodynamic therapy for combined cancer treatment.

## 2. Plasmonic Metal Nanoparticles with Photothermal Effects

Visible or NIR light is primarily employed in most biomedical applications that uses plasmonic nanostructures; therefore, it is crucial to choose the right nanostructures that have an increased absorption of NIR or visible light [69]. Particularly, NIR light (650–900 nm) has been employed extensively in biomedical applications because it can enter deep into the body due to biological tissues’ lower photon absorption and scattering (e.g., blood, water, melanin, and fat) [70,71]. Since their LSPR spans a broad range of visible and NIR ranges, gold, silver, and copper have received the most attention among the materials studied for photothermal effect-based biomedical functions [72,73]. This contrasts with aluminum, platinum, and palladium, characterized by weak and broad LSPR bands in the ultraviolet range [74,75]. 

Owing to its chemical and biological stability, minimal cytotoxicity in biological environments, and a variety of surface functionalization with biological ligands like deoxyribonucleic acid (DNA), proteins, and antibodies, gold is mainly regarded as the ideal noble metal for biomedical applications [76,77]. Silver possesses exceptional optical properties; this includes more extensive extinction, absorption, and scattering cross-sections; thus, silver is able to facilitates more efficient photothermal light-to-heat conversion in comparison to gold [78]. Silver nanoparticles have also been utilized as transducers for photothermal light-to-heat conversion and antibacterial agents [79].

A review by Delille and co-workers summarizes how inorganic nanocrystals of iron oxide, gold, and semiconductor nanocrystals have inherent optical and magnetic properties that place them as good candidates for the detection of cancer, therapy, and imaging [80]. They reported that there has been progress in designing efficient stabilized NPs in biological media, which prevents aggregation due to high-salinity environments and during protein interactions. Polyethylene glycol(PEG), peptoids, and zwitterions are a few polymers that can be used to coat the surface of NPs to reduce the nonspecific protein adsorption on NPs [81,82]. However, they almost certainly only partially prevent the development of a biomolecular corona.

### 2.1. Plasmonic Gold Nanoparticles

Gold has always been considered one of the prominent valuable metals on Earth. The use of gold has expanded over time due to its malleable and chemically inert characteristics [83]. Gold has emerged as a top contender in the fight to improve the medical field by functionalization to produce a more potent and distinctive materials for therapeutic treatment; in fact, a review by Kang et al. concluded that there is a promising future for modified gold nanoparticles to be used in theragnostics [84]. Reducing gold down to the nanoscale leads to a change in its chemical, physical, and optical properties, which presents opportunities to use these materials for different applications [85]. Since Michael Faraday’s initial report in the middle of the 19th century, when he synthesized gold colloidal solutions, AuNPs have been widely used in biomedicine, such as genomics, gene therapy, and plasmonic photothermal therapy (PPTT). These therapies selectively treat cancerous cells and tumors, selectively destroying bacteria and HIV [86,87].

The use of AuNPs in these applications could be ascribed to their unique size de-pendent properties that set them apart from conventional medicines. The free electrons of the AuNPs oscillate in response to light in the electromagnetic spectrum [88]. This phenomenon, referred to as the surface plasmon resonance (SPR), is caused by the resonant behavior of electrons at a specific light frequency [89]. By turning energy into heat, the surface plasmon oscillation can non-radiatively decay. In addition, AuNPs can selectively focus the treatment on a particular area within a biological system, raising optimism for the possible use of AuNPs to act as vessels that can deliver genetic content and drugs to the locations in which they are required [90,91]. While many nanomaterials, including organic nanoparticles like semiconducting polymers [92], metal oxides [93], quantum dots [94], noble metals (such as Au, Ag, Pt, and Pd), and carbon-based materials (such as carbon nanotubes and graphene) [95], have been developed for photothermal therapy, gold nanoparticles have emerged as the leading agents. They have been extensively studied for their advantages, such as high biocompatibility, surface modification and synthesis, and simplicity in controlling optical and physical properties [96,97,98].

Photothermal therapy uses AuNPs because they make it possible for NIR light to produce a photothermal effect in the range 750–1700 nm. The first window spans 750–1000 nm and the second window spans 1000–1700 nm, where water absorption is minimal. This allows for enough light to penetrate the tissues, reach the tumor area, and photothermally destroy it [99]. It has been shown that only gold nanomaterials in certain morphologies such as Au nanorods, Au nanoshells, Au nanocages, and Au nanostars are the only known Au nanostructures that can absorb NIR light [100]. Recent studies indicate that Au bipyramids, Au nanoprisms, Au nanorings, and AuNP assemblies with structures resembling caterpillars can also be used for photothermal applications [101,102,103].

In their study, Ali et al. summarized the use of gold nanoparticles in PPT Sin advanced clinical application. The PEG-coated gold nanorods (AuNRs) reviewed displayed stable and prolonged blood circulation (half-life of 1 h), with no aggregation in key organs (aside from the liver) up to 72 h. The use of cetrimonium bromide (CTAB)-coated AuNRs shows quick blood clearance and buildup at approximately 0.5 h [104].

#### 2.1.1. Gold Nanorods

Small-size AuNRs are efficient PTT agents with tunable aspect ratios (length/width), a significant absorption cross-section, and a narrow absorption spectrum because of the diminished radiation attenuation impact [105]. Due to their remarkable transmembrane transport and diffusion speeds, AuNRs with an elongated morphology can stay in cancer lesions and infiltrate cells more quickly than other gold nanomaterials [106,107]. 

Recently, smaller AuNRs (30 × 7 nm) were synthesized via a seedless, one-spot synthetic approach and were then successfully endocytosed by macrophages [108]. These cells are a biocompatible “Trojan horse” to aid AuNRs in penetrating cancerous lesions and boosting their in vivo PTT efficacy. Studies showed these tiny AuNRs had greater cell uptake and lower cytotoxicity than standard AuNRs (56 × 14 nm) [109]. In vivo PTT studies demonstrated that the temperature of the cancer lesion rose when treated with AuNRs-laden macrophages, rising from 34.5 to 44.3 °C in 1 minute and reaching a temperature of 53.8 °C after 10 minutes at 808 nm irradiation, with 95 % cancer inhibition after two weeks [110]. Their research extensively investigated the potential of cell-specific nanoagent treatments in clinical oncology [111].

#### 2.1.2. Gold Nanospheres

Gold nanospheres (AuNSPs) absorb NIR light non-radioactively by modulating it to absorb visible photons via second harmonic production. Detailed reviews have been presented on the photothermal effect using built-in nonlinear optical features [112,113]. In addition, AuNSPs often aggregate in colloid solutions due to electrostatic contact, which might increase the NIR absorption [114]. However, due to the inhomogeneous broadening in shape and size, which is unfavorable for dissemination, gold aggregates may exhibit a more comprehensive SPR spectral range [115]. AuNSPs exhibit greater tumor uptake than gold nanoshells and nanorods; they can be easily prepared using bioconjugation approaches [116]. For instance, when exposed to pulsed laser irradiation (800 nm), AuNSPs were able to selectively infiltrate cancer cells via conjugation to anti-EGFR antibodies and exert a lethal thermal effect on cancer cells despite the laser energy being 20 times less than that utilized in the therapy without AuNSPs [117].

#### 2.1.3. Gold Nanocages

Gold nanocages (AuNCs), characterized by hollow interiors and porous exteriors, can be readily synthesized by galvanic substitution of chloroauric acid (HAuCl4) and silver nanocubes [118]. Precise control of the quantity of HAuCl4 allowed for the exact adjustment of the SPR peak of AuNCs to the NIR region. With a size of 40 nm for in vivo delivery, these as-synthesized AuNCs had an absorption cross-section that was five orders of magnitude larger than organic fluorophores like indocyanine green [119]. Moreover, these unique structures give AuNCs the capacity to load drugs and a stimuli-responsive release feature that includes pH, temperature, and enzymes, which makes it possible to build high-performance PTT with a lengthy agent circulation period [120,121].

#### 2.1.4. Gold Nanostars

Gold nanostars (AuNSTs) have strong electric field confinement due to the sharp protrusions around the spherical core, which leads to significant dephasing of coherently oscillated surface electrons [122]. This dephasing can be transferred to the atomic lattice to induce exceptional heat flux at the metal–dielectric interface. Furthermore, due to the sharpness of many of their edges, excessive exposure to GNSTs is best avoided to protect normal cells [123,124]. Experimental research on the ideal characteristics and conditions for AuNSTs to perform extremely effective PTT has been made possible by advancements in AuNST synthesis. For instance, solid tumors in vivo and cancer cells in vitro can be heated using a succession of AuNSTs with mean diameters spanning 25 to 150 nm and corresponding SPR peaks between 500 and 1000 nm [125,126,127]. When AuNSTs are internalized by cells and assembled in endosomes, Espinosa et al. [128] discovered, the particle size of AuNSTs and the laser’s wavelength can substantially impact the heating effect in an aqueous dispersion. However, the pertinence of the results was noticeably diminished across both in vitro and in vivo experiments.

#### 2.1.5. Gold Nanoshells

Among the PTT agents developed over the past years, spherically shaped gold nanoshells (AuNSs) with a dielectric core greater than 100 nm have unquestionably played a key role. GNSs are intriguing candidates for cell-mediated nanoagent therapies in clinical oncology because they have distinctive optical and chemical features [128]. By manipulating the thickness of the shell, the maximum SPR absorption wavelength of AuNSs may be selectively controlled to 800–1200 nm because the plasmon hybridization of AuNSs is greater when the thickness is higher and weaker when the thickness is lower [129,130]. Moreover, GNSs can be conjugated to proteins [131], antibodies [132], or ligands [133] to actively target specific cancer cells while passively accumulating in the tumor site through leaky tumor vasculature. Despite these positive traits, it is still challenging to synthesize real GNSs of good quality with the appropriate size, spherical form, and narrow SPR absorption [134].

AuNSs possess efficient drug loading and delivery capabilities due to their spherical core–shell structure; they are thus excellent candidates for multifunctional PTT, which combines chemotherapy, PDT, immunotherapy, and other treatments synergistically [135]. For instance, high 1O2 quantum-yield photosensitizers (PS) Pd[DMBil1]-PEG5000 (linear tetrapyrrole Pd complexes) [136] were easily conjugated to the surface of silica core–gold shell (CS) after being decorated with a thiol functionality, whose safety has been clinically proven [137].

### 2.2. Plasmonic Silver Nanoparticles

Silver nanoparticles have been used as antibacterial agents [138] and in detection and diagnosis platforms [139], tissue repair materials [140], and personal healthcare products [141] due to their outstanding antimicrobial and wound healing characteristics. Silver nanoparticles, however, have recently entered the area of PTT for cancer treatment [142]. Their use is attributed to their higher heat conductivity compared to other metals, low toxicity, ease of synthesis, metabolic nature, and tunable SPR band [143]. In general, silver nanoparticles used in biomedicine have a plasmon resonance of 410 nm and are spherical, which make them unsuitable for deep-penetrated PTT. However, the plasmon resonance can possibly be precisely modified to the NIR domain by creating anisotropic silver nanoparticles like silver nanospheres, nanotriangles, and nanocages [144]. The capability to synthesize MNPs with specific sizes and shapes using several process parameters is crucial to comprehending and predicting their characteristics and behavior under various circumstances [145,146]. However, preparation of well-defined NPs with reproducible size and shape distributions is still difficult.

#### 2.2.1. Silver Nanospheres

Reduction of silver nitrate with hydrazine hydrate (H_2_N_2_O) or sodium borohydride (NaBH4) forms irregularly shaped silver nanospheres (AgNSs) in a range of sizes around 20 nm [147]. These nanoparticles show plasmonic resonance in the 650–1200 nm biologically transparent window and light conversion. This allows for PTT with deep tissue penetration [148]. However, femtosecond irradiation can potentially damage the morphology of single AgNSs [149]. Thus, organic compounds can be altered on the surface to protect AgNSs.

#### 2.2.2. Silver Nanotriangles

The plasmonic resonance of silver nanostructures may be tuned into a triangular form, which gives the particles a great deal of potential as NIR-responsive photothermal agents [150]. Applying biopolymers—such as chitosan—for coating is essential to stabilize silver nanotriangles (AgNTs), avoid self-aggregation, and mitigate their cytotoxicity by preventing the release of silver ions [151]. This is because the corners of these AgNTs are susceptible to oxidation, which can lead to a blueshift in the absorption and reduce the photothermal effect [152].

#### 2.2.3. Silver Nanocages

Other silver nanoparticles have been prepared using the peptide template octreotide to facilitate the synthetic process. Bian et al. [153] prepared hierarchical mineralized silver nanocages. The hollow nanoshell structure of the silver nanoparticles, with extremely strong plasmonic coupling, results in silver nanocages that shows significantly increased SPR and NIR absorption above 900 nm, which is useful in the therapeutic window for PTT [154]. Research revealed that silver nanocages’ power conversion efficiency (PCE) reached 46.1%, and a tumor-killing efficacy of over 82.7 % was attained [155]. Using a biological template rather than a harmful surfactant, the silver nanocages’ undetectable toxicity was transmitted at the treatment dose [156].

## 3. Functionalization of Inorganic Nanoparticles

A simple general principle can be used to examine the surface chemistry of inorganic NPs: (i) to achieve water solubility, surface ligands must interact with the NP’s inorganic surface [157] and (ii) have hydrophilic groups [158]. However, surface ligand molecules can exist during the synthesis of AgNPs in aqueous media. These ligands are typically unsuitable for direct in vivo applications since they are needed to govern growth, size, and shape [159,160]. In contrast, it is uncommon to find surface ligands suitable for in vivo applications and for the direct water synthesis of NPs. As a result, NPs are more frequently prepared using a group of starting ligands [161]. When the original ligands are hydrophobic, the NPs can be encapsulated with amphiphilic compounds or polymers to transfer to aqueous media. The final desirable hydrophilic surface chemistry can also directly react with the initial ligands [162,163].

### 3.1. Functionalization of Silver Nanoparticles

Every application requires appropriate surface functionalization strategies, since the functional groups influence the colloidal stability of the nanoparticles. These can facilitate either the controlled assembly or the targeted delivery of the as-prepared nanoparticles, [164,165]. Surface functionalization of nanoparticles with biomolecules modifies the material’s surface composition, structure, and morphology while maintaining the overall mechanical properties [166]. Additionally, surface enhancement of nanomaterials is crucial because it decreases surface energy while acting as a barrier that prevents nanoparticles from aggregating together and decreasing their long-term impact [167]. Metal nanoparticle synthesis, manipulation, and organization are all possible using various techniques. The most often used approaches include deposition of the particles on structured surfaces, by incorporation of the particles onto glassy surfaces, through leverage of the biomolecules as linkers using bivalent linker chemicals [168,169].

Studies using bare AgNPs as plasmonic biosensors are quite scarce, even though most biosensors function ex vivo because of their toxicity. A detailed account of AgNP toxicity was published in a book in 2019 [170]. The lack of stability and complicated surface chemistry of bare AgNPs is the second factor and most likely the main restriction for their usage in bio-applications [171,172]. AgNPs can be coated with a wide range of substances to get around these restrictions; the coating procedure significantly affects the trajectory, toxicity, and stability of AgNPs in a specific environment [172]. The NP coating creates electrostatic, steric, or electrosteric repulsive forces between the particles, preventing them from aggregating [173].

Among the most extensively studied polymers for stabilizing or coating NPs is poly(ethylene) glycol (PEG) [174,175]. The Food and Drug Administration (FDA) has authorized the application of this neutral, hydrophilic, and biocompatible polymer for biomedical and pharmaceutical purposes [176]. By sterically hindering AgNPs and preventing nanoparticle aggregation, PEG increases AgNPs’ dispersity in physiological settings. Reaction 1 below shows one of many environmentally friendly techniques to encapsulate AgNPs with PEGs. AgNPs with PEG coating stabilize colloidally, most likely as a result of van der Walls (VdW) interactions:

$${{\mathrm{Ag}}^{+}}_{\left(\mathrm{aq}\right)}+{\mathrm{PEG}}_{\left(\mathrm{aq}\right)}\to {\left[\mathrm{Ag}(\mathrm{PEG})\right]}_{\left(\mathrm{aq}\right)}^{+}$$

(1) $$2\left[{{\left[Ag\left(PEG\right)\right]}^{+}}_{\left(aq\right)}+{CH}_{2}OH{\left({CH}_{0}H\right)}_{4}CHO⟶2{\left[Ag\left(PEG\right)\right]}_{s}+{CH}_{2}OH{\left(CHOH\right)}_{4}COOH\right.$$

*Van der Waals* forces fulfil a crucial contribution in the stabilization of silver nanoparticles (AgNPs) if PEG has been attached to their surface [177]. These forces, which involve dipole–dipole interactions, dispersion forces, and dipole-induced dipole interactions, enable the attraction of PEG molecules and AgNPs [178]. When PEG molecules coat the surface of AgNPs, van der Waals forces facilitate a uniform and dense coating of the nanoparticles. This dense PEG layer triggers steric hindrance, preventing nanoparticles from colliding and aggregating [179]. The homogeneous PEG coating also attracts water molecules, generating a hydration shell around the nanoparticles, improving their stability in aqueous media. The amalgamation of these attractive forces keeps the PEG chains firmly bonded to the AgNPs, resulting in a stable colloidal suspension. By steering clear of aggregation via these van der Waals interactions, PEGylated AgNPs remain well dispersed and stable [180]. This is critical for their successful application across diverse biomedical applications, comprising drug delivery, imaging, and treatment.

Excellent optoelectronic characteristics are a well-known characteristic of silver nanoparticles [181]. These distinctive visual characteristics result from a group of conduction electron oscillations known as SPR. Below are some factors attributed to these oscillations:

- The enhancement of the electrons in the electric field of the incident radiation, which is one of the mechanisms that causes these oscillations;
- The existence of restoring forces brought on by the induction in the polarization of the particle and the medium around it;
- The confinement of the electrons to specific dimensions.

These attributes are impacted by the particles’ size, shape, composition, environment, and spatial arrangement. Extensive research has been conducted to improve photothermal therapy for bacterial infections and cancer [182]. This section examines some studies conducted to functionalize AuNPs and AgNPs. One of the most researched methods to address bioavailability and selectivity issues is the conjugation of anticancer drugs into vehicles/delivery tools. These characteristics are influenced by the particles’ size, shape, composition, environment, and spatial arrangement. Numerous studies have been conducted to improve photothermal therapy for bacterial infections and cancer [183,184,185]. 

One of the ways to functionalize AgNPs is by using biomolecules. Biosensing, imaging, and hyperthermia therapy are biological applications made possible by the guided interaction of proteins with different kinds of nanoparticles [186,187]. The approach yields an appropriate interface for actual biological systems’ surroundings. There are four main groups of techniques for coupling biomolecules to nanoparticles:

1. The biomolecule’s attachment to the inorganic particle’s surface via ligand-mediated binding, frequently by chemisorption, for instance, thiol groups, to the core;
2. Positive charges interact electrostatically with negatively charged nanoparticles to biomolecules or the other way around versa;
3. Covalent bonding by conjugation chemistry, utilizing groups focused on both biomolecules and particles;
4. Receptor–ligand systems are affinity-based but non-covalent.

An ideal surface functional group is necessary for nanoparticle binding with different biomolecules. A great diversity of organic molecules in nature provides distinct biological processes and microorganisms’ form and function [188]. Such molecules range in composition, size, and complexity. On the one side, there are small particles like lipids, vitamins, peptides, and carbohydrates; on the other, there are larger molecules like natural polymers, proteins, enzymes, DNA, and RNA [189]. Since the surfaces of many biomolecules contain alcohol, phosphate, primary amine, carboxylic acid, or thiol groups, an array of molecules can be bonded to the surface of a metal nanoparticle to make it functional.

### 3.2. Functionalization of Gold Nanoparticles

Early findings on Au nanostructures focused mostly on visible-light-absorbing Au nanospheres, while more recent research has concentrated on increasing their plasmonic absorption to the NIR window [190]. As demonstrated, it has been observed that an increase in the size and forming anisotropy of Au nanostructures is an efficient technique to move the plasmon absorption band from the visible up to the near-infrared spectrum [191,192]. Another variable that may be adjusted to modify the plasmon-induced optical response and obtain significant NIR absorption is the ligands [193]. 

**Figure 5:**
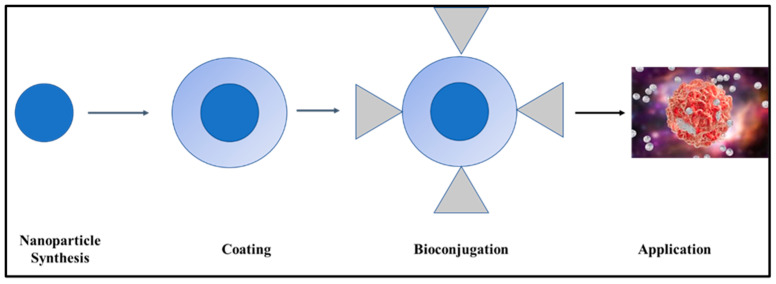
Steps generally used for functionalizing nanoparticles.

The following actions must be prioritized to use functional NPs for biosensing and bioimaging, as shown in Figure 5: (a) synthesis, (b) coating, (c) surface functionalization or bioconjugation, and (d) applications. To enhance the solubility and stability of NPs in aqueous media, functionalization (surface modification) is one of the necessary processes (hydrophilicity). This improves their original qualities, which are crucial for biomedical applications, and boosts their biocompatibility and biofunctionality [194].

A unique advantage of AuNPs in bioassays is the ability to tune particle sizes precisely [195]. Additionally, it is simple for biomolecules with exposed thiol groups to form gold–sulfur bonds and bind to the gold surface [196]. A biofunctional molecule is typically used, with its carboxyl/amine termini exposed for conjugation with functional entities and its thiol termini immobilized on the particle surface [197]. Amid the reduction of gold salts, bi-functional thiol molecules were attached to Au nuclei. These NPs are commonly designated as monolayer-protected clusters [197]. The surface of AuNPs is modified with various biofunctional groups, such as proteins, nucleic acids, and amphiphilic polymers, creating a suitable foundation for the application of functionalized AuNPs for biofunctionality [198].

### 3.3. The Use of Polymers for Biofunctionality

Due to their great biocompatibility and simple surface functionalization, polymers are essential in designing, manufacturing, and developing multifunctional nanoparticles [199,200]. Polymeric materials, among other multifunctional nanomaterials, have much promise as an innovative approach to treating human ailments. The three main categories of the encapsulating approach utilized to create the polymeric multifunctional nanoparticles are layer-by-layer, solvent evaporation, and polymerization coated [201,202]. PEGylated AuNPs demonstrate improved stability and solubility. AuNPs can be prepared using surface sputtering in different solutions, for example, (i) sodium citrate dihydrate (TCD) [203] and (ii) N-acetyl-L-cysteine (NALC) with the addition of PEG. Further surface grafting can be carried out on the nanoparticles using polyethylene naphthalate (PEN) [204]. These nanoparticles showed improved stability and optical properties.

Colloidally stable AuNPs were prepared using thiol-functionalized ionic liquids as stabilizers [205]. Thiol groups may be either attached to the cation or anion and have symmetrical or unsymmetrical positions. They were designed to fit the features of the ionic liquids [206]. Au-S is the primary form in which polymers and AuNPs are covalently connected. To evaluate the inhibitory action of polyacrylic acid (PAA)-coated AuNRs on human osteosarcoma cells in vitro, Pan and co-workers synthesized them [207]. They then described AuNRs as prospective candidates for phototherapeutic applications in human osteosarcoma. Their findings showed that AuNRs@PAA caused DNA integration and disrupted cell membranes, which led to cell death in human osteosarcoma cells. Nanorods have been used in vivo and in vitro as photothermal therapeutic agents [208].

Both in vitro and in vivo experiments on the effective usage of nanocages (AuNCs) as photothermal therapeutic agents have been published. For instance, Hong and colleagues [209] created NIR-absorbing AuNCs and functionalized them with PEG and the AS1411 aptamer to produce cancer-targeted PTT (AS1411-PEG-AuNCs). They showed that when exposed to NIR light, AS1411-PEG-AuNCs killed malignant cells only.

#### The Effect of Surface Modification of the Plasmonic Nanoparticles

Nanoparticles’ surface parameters, such as charge, hydrophobicity, and functional groups, impact how they interact with proteins in the bloodstream [210]. Upon entering the body, nanoparticles are shortly enveloped by a layer of serum proteins, which might alter their biological identity and behavior [211]. For example, nanoparticles with hydrophobic surfaces or positive charges tend to adsorb more serum proteins due to hydrophobic interactions and electrostatic attraction [212]. This protein corona can potentially impact nanoparticle biodistribution, cellular uptake, and clearance. Surface modification with hydrophilic polymers, for example, PEG, can minimize protein adsorption, resulting in a stealth effect that increases circulation time and reduces immune detection [213].

Surface changes have an impact on nanoparticles’ hydrodynamic diameter and zeta potential. Surface modification can considerably increase the hydrodynamic diameter, including the nanoparticle core and any surface-bound molecules or protein corona [214]. For example, including a PEG layer often improves the hydrodynamic diameter. Surface changes can affect the zeta potential, which reflects the surface charge of nanoparticles, allowing for desired interactions with biological systems. A neutral or slightly negative zeta potential can limit nonspecific interactions and aggregation, improving stability in biological fluids [215]. Conversely, strongly positive or negative zeta potentials can encourage aggregation and alter the cellular uptake [216]. Additionally, surface changes can affect phagocytosis, the process by which immune cells engulf and eliminate nanoparticles. Surface modifications such as PEGylation, which create a hydrophilic and neutral surface, can reduce opsonization, which is the marking of nanoparticles for phagocytosis and subsequent absorption by phagocytic cells, extending the nanoparticles’ presence in the bloodstream and improving their ability to deliver to specific tissues [217].

## 4. Application of Plasmonic Nanoparticles

Plasmonic nanoparticles have been pervasively applied for their light scattering as nanoantenna or contrast agents for surface-enhanced Raman scattering [218], metal-enhanced fluorescence [219], and optical imaging such as dark-field and computed tomography [220]. This is attributed to their larger optical cross-sections, as opposed to organic dyes, commonly used for bioimaging and sensing. In addition to radiative scattering, nanoparticles can absorb light non-radiatively, which can produce considerable heat energy or photoluminescence [221,222]. Particularly, photothermal therapy applications have substantially utilized plasmonic nanoparticles’ ability to convert light to thermal energy, referred to as the photothermal effect [223,224].

Photothermal therapy (PTT) utilizes hyperthermia, a less invasive cancer treatment, and has received widespread approval [225]. However, it is rarely used in clinical practice due to insufficient tumor selectivity and the challenges in heating deep tumors to therapeutic temperatures. By utilizing MNPs to specifically target tumor cells, advancements in nanomedical research have overcome these restrictions [226]. Plasmonic photothermal therapy is derived from metal nanoparticles strongly absorbing electromagnetic energy when delivered and transformed into heat through electron excitations and relaxations (PTT) [227].

**Figure 6:**
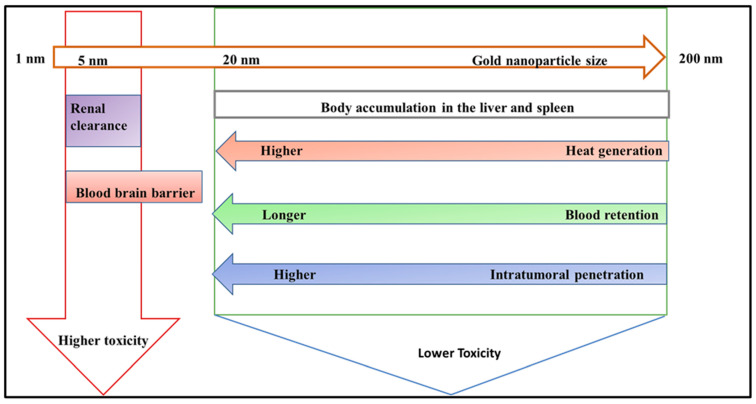
The effect of AuNP size on their biological behavior. Smaller-sized nanoparticles result in a high clearance from the liver and the blood–brain barrier.

The AuNPs should ideally build up inside the tumor for PTT to succeed. However, the size, surface coatings, and routes of administration of AuNPs all play a crucial role in the distribution and pharmacokinetics of AuNPs in the body [228]. Generally, the reticuloendothelial system (RES), mediated by macrophages in the liver and spleen, is the primary clearance mechanism for nanoparticles injected intravenously [229]. The blood circulation duration increases due to the reduced contact between nanoparticles and the RES, and this prolonged time is frequently correlated with more intratumoral penetration [230]. The enhanced permeation and retention (EPR) effect, directly linked to immature and leaky tumor blood capillaries, causes nanoparticles to collect in the tumor [231]. Additionally, nanoparticles must pass a barrier surrounded by dense stromal tissues and under high interstitial fluid pressure to enter the tumor [232]. It might be more advantageous to use smaller AuNP sizes to get around these obstacles. Figure 6 summarizes the impact of AuNP size on toxicity, clearance pathways, heat generation effectiveness, blood circulation speed, and intratumoral penetration capacity. Smaller AuNPs (>20 nm) would be more favorable for PTT. 

Hawash et al. synthesized novel 3-methyl-4-phenyl-isoxazole-Carboxamide derivatives for melanoma and a nano-emulgel conjugate to increase cellular permeability. The synthesized products were proven to have significant oral bioavailability. Additionally, the drug score was calculated and revealed that the majority of the synthesized products had good drug scores (0.31–0.61), indicating good drug-likeness performance [233].

Size significantly affects the distinctive properties of AuNPs, including heat generation, blood retention, and intratumoral penetration [234]. Smaller gold nanoparticles exhibit an increased surface area-to-volume ratio, increasing their photothermal conversion efficiency [235]. This means they can absorb light more effectively and convert it to heat, making them ideal for applications like photothermal therapy. Furthermore, smaller nanoparticles elude rapid clearance by the mononuclear phagocyte system, resulting in longer blood circulation durations. This prolonged blood retention increases the possibility of nanoparticles collecting in tumors owing to the enhanced permeability and retention (EPR) effect, which is critical for effective cancer treatment [236]. Additionally, given that these nanoparticles are smaller, they can penetrate deeper into the tumor microenvironment and reach cancer cells that larger particles cannot. This improved intratumoral penetration is crucial for delivering therapeutic drugs directly to the target site, increasing therapy efficacy. The smaller size of gold nanoparticles improves their functional properties, making them ideal for medical applications that need precision targeting and efficient therapeutic action [100].

In their work, Qiu et al. [237] successfully developed a dual-functional localized surface plasmon (LSPR) biosensor by their combination of the plasmonic sensing transduction and the photothermal effect for the identification of SARS-CoV-2 nucleic acid. For sensitive and precise severe acute respiratory syndrome coronavirus 2 (SARS-CoV-2) detection, the plasmonic chip with the two-dimensional distribution of nano-absorbers (AuNIs) produced local PPT heat and transduced in situ hybridization. This dual-functional LSPR biosensor that has been developed can offer a reliable and simple-to-use diagnostics platform to raise the diagnostic precision of clinical testing and lessen the burden on PCR-based tests [238]. A review by Lv and his colleagues [239] highlighted the recent development that one-spot seedless synthetic techniques could be used to create small GNRs (30 nm–7 nm), which were then successfully endocytosed by macrophages. These cells are a biocompatible “Trojan horse” to aid AuNRs in infiltrating cancer lesions and increasing their in vivo PTT efficacy.

### Toxicity and Biodegradability of Metal Nanoparticles

MNP toxicity and biodegradability are essential concerns for their application in cancer treatment by PDT and PTT. While MNPs have distinct advantages, such as efficient light absorption, high photothermal conversion efficiency, and the ability to produce reactive oxygen species (ROS), their potential toxicity can provide considerable hurdles. Toxicity concerns stem primarily from the persistence of MNPs in the body and their ability to produce oxidative stress, inflammation, and damage to healthy tissues and organs [240]. For example, AuNPs and AgNPs may accumulate in crucial organs such as the liver, spleen, and kidneys, causing long-term damage. Furthermore, non-degradable MNPs may cause persistent exposure, exacerbating the adverse consequences [241].

To address these issues, researchers have attempted to develop MNPs that are both efficacious and biodegradable. Biodegradable MNPs can be designed to degrade into non-toxic metabolites readily eliminated from the body. For example, iron oxide nanoparticles (IONPs) are thought to be more biocompatible because they can be metabolized into iron ions, which the body naturally regulates. Furthermore, surface changes can improve the biocompatibility of MNPs [242]. By coating them with biocompatible polymers or natural compounds like chitosan, MNPs can be less toxic and more stable in biological contexts. Another technique entails using composite nanoparticles that blend biodegradable elements with metal cores, preserving the therapeutic benefits of MNPs while reducing their toxicological impact [243]. Furthermore, specific control of MNP size, shape, and surface charge can affect their dispersion, cellular absorption, and clearance, reducing potential toxicity.

## 5. Progress in Photodynamic Therapy

Photodynamic therapy serves as a therapy that causes minimal invasion of “normal” cells in the treatment of antimicrobial resistance (AMR) infections and cancer [244]. For the PDT process, there are mainly three requirements: oxygen, photosensitizer, and light of a specific wavelength. Combining these factors produces lethal cytotoxic effects to destroy tumor cells [245,246]. A photosensitizer is a drug that preferably localizes on a diseased cell and is activated by light of a defined wavelength in the presence of molecular oxygen to produce cytotoxic species such as triplet oxygen or radicals [247]. In PDT, unlike many other treatment methods, the application can happen multiple times at the same site without compromising the circumferential tissues. PDT also serves as an advantage, as it is utilized for large, buried tumors and any extra microscopic diseases omitted by other treatments such as surgery, and this is because it uses light that is directly fed to the tumor [248].

Generally, the red region in the visible spectrum is where human tissue transmits light most effectively [249]. This means that photosensitizers with 650–800 nm absorption are excellent photosensitizers to penetrate deeper tissues. Porphyrins have been one of the excellent potential photosensitizers researched compared to other dyes. This is because of their low toxicity and because they undergo no detectable metabolic alterations without chelated iron ions [250]. Porphyrins have a bile–gut pathway as their clearance from the organism [251]. Porphyrins containing no centrally coordinated ions exhibit an absorption band in the wavelength region above 600 nm. This means their photoactivation is by the illumination with light in the red spectral region [252]. Photofin, which is a combination of porphyrin dimers and higher oligomers where the porphyrin units are conjugated by an ether, ester, and carbon–carbon bonds, has been authorized in different countries, including Canada, Europe, and Japan, for the treatment of various AMR infections [253,254]. Photofins are excellent photosensitizers; however, they impose certain restrictions, such as the photosensitivity of the skin, relatively low absorption at long wavelengths, and little optimal light to penetrate tissues and go in greater depth [255].

Second-generation photosensitizers are designed to overcome the weaknesses of first-generation photosensitizers. Photosensitizers include benzoporphyrins [256], phthalocyanines [257], and purpurins [258], and these were developed in various laboratories around the world. The third generation of photosensitizers is also being developed to enhance second-generation photosensitizers [259,260]. The two main study points for this are gene engineering and the use of nanotechnology in PDT. Porphyrins are 22 π electron systems with the main aromatic conjugation pathway containing 18 π electrons, so they have long wavelengths and an intense color associated with them [208]. Most porphyrinoid photosensitizers have multiple absorption bands to allow for physical tissue depth and regulated penetration. Porphyrins have an optical spectrum with a strong transition (π-π*) around 400 nm (Soret band) as well as four Q bands in the visible region [261].

**Figure 7:**
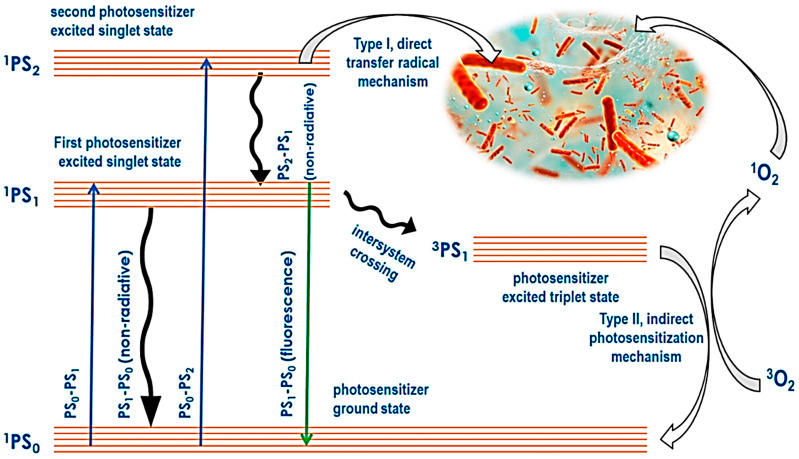
Scheme that shows how a photosensitizer is activated and the production of singlet oxygen leading to cell death.

PDT uses a conjunction of molecular oxygen, photosensitizers, and light to target, resulting in cytotoxic activity selectively. The tumor cells and macrophages have preferential uptake of the photosensitizers, which are activated by light. The photosensitizers then become excited and take a triplet form, and their reaction with molecular oxygen produces reactive oxygen species (ROS) [262]. The hydroxyl radicals also induce the reaction between the molecular oxygen and the photosensitizer. The cytotoxic molecules produced lead to a series of biological reactions that eventually lead to cell death. Figure 7 below summarizes the mechanism of PDT.

### 5.1. Type I and Type II Photodynamic Therapy

PDT includes two principal mechanisms: Type I and Type II routes, which both include the activation of a photosensitizer by light in the presence of oxygen to cause cytotoxic effects. In Type I PDT, the activated photosensitizer directly interacts with biological components such as proteins, lipids, and nucleic acids, producing free radicals and other reactive oxygen species (ROS) via electron or hydrogen transfer mechanisms [263]. These free radicals can cause significant cellular damage by damaging various cellular structures and biomolecules, ultimately leading to cell death. Type I reactions are less dependent on oxygen and can occur even in hypoxic situations, making this route particularly advantageous for treating tumors with low oxygen levels [264].

In contrast, Type II PDT includes the interaction of an excited photosensitizer with molecular oxygen to generate singlet oxygen (^o^), a highly reactive form of oxygen. Singlet oxygen predominantly causes oxidative damage to cellular membranes, organelles, and other essential components, eventually leading to cancer cell apoptosis or necrosis [262]. Type II PDT depends on oxygen availability, as singlet oxygen production requires adequate oxygen levels in the tumor microenvironment. The dependence on oxygen may limit the efficacy of Type II PDT in hypoxic tumors. However, Type II PDT is considered more selective and efficient for generating singlet oxygen, which is extremely powerful in activating cell death [265]. The balance of Type I and Type II processes varies depending on the photosensitizer utilized, the oxygen concentration in the tumor, and the local cellular environment.

### 5.2. Hypoxia Targeting for Cancer Treatment

Hypoxia targeting with metal nanoparticles is an innovative cancer therapeutic technique that uses a specific microenvironment of solid tumors. Hypoxic areas of tumors have low oxygen levels and are frequently resistant to traditional therapies such as chemotherapy and radiotherapy [266]. Metal nanoparticles provide a diverse platform for hypoxia targeting due to their distinct physical and chemical features. In addition to the leaky vasculature and inadequate lymphatic drainage common in tumors, MNPs accumulate more in tumor tissues than in normal tissues through passive targeting via the enhanced permeability and retention (EPR) effect [267]. The size and structure of MNPs can be modified to maximize their accumulation in hypoxic tumor locations via the EPR effect. Active targeting uses hypoxia-responsive components such as hypoxia-inducible factors (HIFs), which are transcription factors that activate under low oxygen circumstances [268]. MNPs can be modified with ligands that bind specifically to HIFs or hypoxia-inducible proteins, allowing for targeted therapy.

PDT and PTT can also serve as effective hypoxia treatments [268]. When exposed to light, MNPs can transport photosensitizers to produce reactive oxygen species (ROS) that may cause cell death. Hypoxia-specific photosensitizers are being developed to improve PDT efficacy in hypoxic tumors [269]. For PTT, MNPs can transform light energy into heat, which results in localized hyperthermia and cell death. AuNPs and other MNPs with high plasmonic characteristics are helpful in this application. A variety of metal nanoparticles are employed for hypoxia targeting. AuNPs are easily functionalized with hypoxia-responsive ligands, drugs, or targeting moieties, and their high plasmonic resonance makes them ideal for imaging and PTT [270]. AgNPs are naturally antibacterial and can be manipulated for targeted drug administration and imaging [138]. External magnetic fields can guide iron oxide nanoparticles (IONPs) to tumor locations, increasing their concentration in hypoxic regions. They are also used in imaging (as MRI contrast agents) and therapy (magnetic hyperthermia) [271]. Titanium dioxide nanoparticles (TiO_2_ NPs) have photocatalytic activity can yield ROS under UV light, rendering them appropriate for PDT. They can also be functionalized with hypoxia-responsive components for targeted drug delivery [272].

Porphyrin aggregation can impact their photodynamic efficiency, resulting in diminished therapeutic efficacy in PDT. When porphyrin molecules aggregate, their proficiency to generate reactive oxygen species (ROS) upon light activation is reduced [273]. This is primarily because aggregation causes a quenching effect, in which the excited singlet and triplet states of porphyrin molecules are deactivated via non-radiative routes rather than generating ROS [274]. Moreover, aggregation can change the absorption properties of porphyrins, altering their spectra and reducing overlap with the therapeutic light source. This spectrum shift complicates the activation of the photosensitizer and its subsequent photodynamic action. Furthermore, aggregated porphyrins might have altered biodistribution and decreased cellular absorption, limiting their concentration at the target site [275]. As a result, maintaining the monomeric form of porphyrins is critical for maximizing photodynamic efficiency and achieving successful PDT results.

### 5.3. Combination of Methods, PDT, PTT, and Magnetic Hyperthermia (MH)

One disadvantage of PDT is that it results in the photosensitizer staying in the patient’s body for longer periods, making it available for the patient to be more sensitive to light. PTT is an alternative to PDT. In PTT, there is partial precise heating of the local environment. Upon light absorption, the PTT agents will cause transitions from the ground state to the excited state [276]. Subsequently, the energy from the electronic excitation relaxes different non-radiative decay channels. The kinetic energy increases, leading to overheating the local environment around light-absorbing species [277]. Heat production, in turn, destroys the cells and tissues (local). Metal nanoparticles are excellent candidates as PTT agents due to enhanced absorption cross-sections, which have been proven to be five or four magnitudes larger than those by photo-absorbing dyes [278]. Due to the strong absorption by metal nanoparticles, there will be laser therapy at relatively low energy, which means that the therapy resulting from that will be less invasive. The nanostructures of metals exhibit a higher photostability and do not suffer photobleaching [279].

Another major PDT challenge is delivering hydrophobic porphyrins to the target sites. Many nanoparticles accumulate rapidly in solid tumors through enhanced permeation and retention (EPR). This results from a compound of many things like the deficiency in lymphatic drainage, leaky vasculature, and an increased impermeability of the vessels [280]. The encapsulation of porphyrins with other nanoparticles increases their suitability for tissue delivery, and this phenomenon creates hydrophilicity, immune tolerance, specific tissue lifetime, and targeting [281]. Nanoparticles also allow for the combination of PDT with other therapies, such as radiotherapy and hyperthermia [282]. Due to basic issues connected with local hyperthermia, such as heterogeneous temperature dispensation in tumor mass and difficulty in regulating the overheating at the deep-seated tumor location, hyperthermia could have been more helpful in treating malignant cells [283]. As a result, a novel technique must be developed to address these critical issues. On this occasion, scientists have proposed nanotechnology as a therapy option that is both safe and effective. Using MNPs demonstrates that heat will be generated to increase hyperthermia efficiency [284].

## 6. The Use of Plasmonic–Magnetic Nanohybrids

There has been a growing interest in using plasmonic–magnetic nanohybrid systems for cancer therapy. Some researchers have actively used the spinel ferrite family to explore the magnetic component [285]. In their work, Qiu et al. [286] synthesized a functional nanohybrid composed of a plasmonic Au core and a magnetic MgFe_2_O_4_ shell. They reported that in vivo studies showed that the prepared Au@MgFe_2_O_4_ nanohybrids showed photothermal therapeutic effects because they could annihilate cancer cells in tumor-bearing mice under the NIR illumination. The substantial darkening of the tumor location following the delivery of the hybrid showed that the nanohybrids have a saturation magnetization value sufficient for efficient T_2_-based MR imaging [287].

In their study, Limon et al. [254] synthesized water-soluble AuNP, which can act as carriers for peptides that may have an anticancer effect but whose bioavailability is constrained by physicochemical factors, including high molecular weight and limited water solubility. They developed peptide BPC734 in their research, which has the same peptide backbone as BPC194 but only one available amino group that can be conjugated to the carboxylic groups of the nanoparticles. A 1-(4,4-dimethyl-2,6-dioxocyclohex-1-ylidene)-3-methylbutyl (ivDde) group protects the remaining amino groups. Therefore, it is anticipated that these non-polar protecting groups will alter the peptide’s physicochemical characteristics and bioavailability, which will affect its cytotoxicity. An alteration of the previously known Brust–Schiffrin synthesis technique, consisting of the reduction of an aqueous gold (III) solution by sodium borohydride with thiol-bearing ligands, was employed to produce gold nanoparticles of an average size of 10 nm [288]. A combination of two distinct thiol linkers was chosen for this design, one of which contains polyethylene glycol and a terminal hydroxyl group (PEG.OH) to provide hydrophilicity, and the other of which is similar but has a terminal carboxylic acid group (PEG.COOH) to provide water-solubility and enable peptide conjugation [289]. By employing this technique, gold nanoparticles (AuNP.OH/COOH) highly soluble in water were synthesized, and UV-VIS absorption spectroscopy revealed an absorption band at 523 nm, which corresponds to a distinctive surface plasmon resonance (SPR) band of AuNP. In the presence of N-Hydroxysuccinimide (NHS) and 1-Ethyl-3-(3-dimethylaminopropyl) carbodiimide (EDC) at pH 7.4, BPC734(cyclic peptide) was subsequently covalently conjugated to AuNP by forming an amide bond observed between its free amino group and the carboxylic groups on the nanoparticles [290].

### 6.1. The Decoration/Capping of Gold and Silver Nanoparticles with Porphyrins

Fabricating dye molecules with defined photophysical properties is mandatory for multiple purposes: bioimaging, photocatalysis, and optoelectronics [291,292]. Based on a detailed study of the correlation between the photophysical structure and properties of such dyes, many methods for modifying their properties have been investigated [293,294]. Despite these efforts, it is still difficult to precisely modify dye qualities inherent to their electronic structure because the molecular design technique only allows for incremental modifications. Conjugating dyes and metal nanoparticles is one of the various methods for modifying the photophysical characteristics [295,296]. The MNPs and porphyrin nanoconjugates are summarized in Table 1 below.

**Figure 8:**
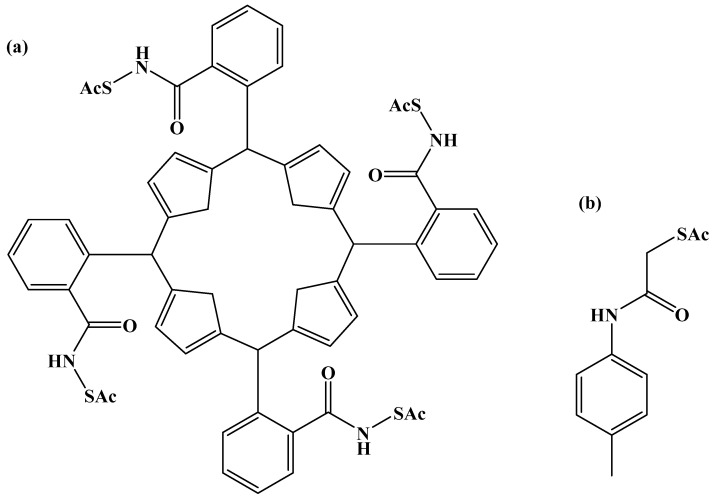
Porphyrin surface-protected ligands (**a**) and (**b**) ligand.

Organic compounds, especially thiol and disulfide derivatives, have been used to protect ligands for gold nanoparticles to control the size of gold nanoparticles effectively [297]. Gold nanoparticles with thiolate shielding can be synthesized by reducing HAuCl_4_ with NaBH_4_ in the presence of thiol or disulfide derivatives [298]. The size of gold nanoparticles can be controlled by manipulating the molar ratio of thiol (or disulfide) to HAuCl_4_. The etherification of PEGylate porphyrin groups to form the AuNP@5,10,15-p(ῳ-methoxypolyethyleneoxyphenyl)-20-p(hydroxyphenyl) porphyrin (AuNP@Porf@PEG) nanosystem after the surface functionalization of gold nanoparticles using a 3-chloro-1-propanethiol monolayer, which preserves the free methyl chloride functionality [299,300]. By using rigid linkers that point in the same direction and are perpendicular to the porphyrin plane, it is possible to design a novel tetradentate porphyrin protective ligand with four sulfur atoms as shown in Figure 8.

Due to their exceptional dispersibility, gold nanoparticles (AuNPs) are frequently protected with alkanethiol groups, as established by Yang et al. [301]. Over the past 25 years, they are frequently used for this purpose. By using thiol/thiolate exchange processes, the alkanethiolate adsorbates can easily be swapped out for different thiolates to produce AuNPs that have been altered by entering adsorbates. Multifunctional nanoparticles can be produced through consecutive or simultaneous adsorbate exchange reactions by adding or changing desirable functional characteristics, as exemplified by solubility, charge, and affinity with other molecules. Changing the size, shape, and length of the linkers and nanoparticles is the general approach for adjusting the photophysical characteristics of dye–AuNP conjugates [302,303].

**Figure 9:**
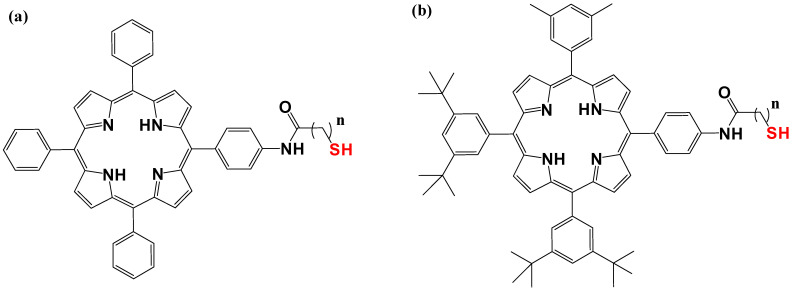
Porphyrin–alkanethiols. (**a**) tetraphenyl porphyrin-5-yl) phenyl) acetamide, (**b**), 5,10,15 3,5-di-tert-butyl(phenyl) porphyrin-5-yl)phenyl)acetamide.

**Figure 10:**
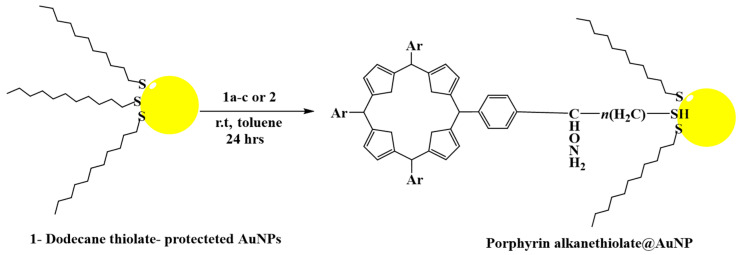
Porphyrin–alkanethiol post-synthesis loading on 1-dodecanethiolate-protected AuNPs.

By using 1-dodecanethiolate-protected AuNPs as the precursors for post-synthetic thiol/thiolate exchange processes, porphyrin-AuNP conjugates were synthesized with adsorbate loading levels of up to 10% of all accessible thiolate sites [304]. After loading, the conjugates maintain the distinctive photo-absorption properties of porphyrin (the Soret band and Q band). The Soret band showed a slight red shift, shoulder expansion, and broadening, while the Q bands did not. Notably, porphyrin adsorbates quickly produce H-aggregates after starting the thiol/thiolate exchange process [305]. Figure 9 and Figure 10 below show examples of chemical structures of porphyrin–alkanethiols.

Numerous ligand-functionalized metal nanoparticles have been discovered based on ligation. These nanoparticles are stabilized by the chemical attraction of organic functional groups to the surface of the nanoparticles [306]. Using the σ electrons of the functional groups, polymers, linear molecules with long alkyl chains, and dendrimers have all been successfully exploited for this intention [307]. Functionalization can also be through the formation of Au-strong multidentate ligation using thiol derivatives to obtain Au nanoparticles surrounded by π orbitals [308]. The multidentate macrocyclic porphyrin thioester derivatives tetrakis-5,10,15,20-(2-acetylthiophenyl)porphyrin (SC_0_P) and tetrakis-5,10,15,20-(2 acetylthiomethylphenyl)porphyrin (SC1P) were synthesized and designed by the insertion of methylene groups between the benzene and the acetylthio groups to enhance the separation between the porphyrin ring and the surface of the Au [309].

Developing functional nanocomposites with controlled structural properties is best accomplished by designing organic–inorganic hybrid molecular materials from the bottom-up [310,311], because of its optical properties methods. Varied particle sizes and shapes lead to various optical characteristics, primarily the surface plasmon appearing at various wavelengths. In contrast to Mie’s prediction, the surface plasmon exhibits a blueshift for AuNPs smaller than 50 nm. The wavelength of the surface plasmon may also be significantly affected by additional structural parameters, including aggregation and shape irregularity [312,313]. AuNPs with a few atoms can be considered big molecules with distinct energy levels. Their luminescence adheres to the free-electron model and results from sp-sp electronic transitions rather than sp-d transitions. Unfortunately, a drawback of these molecular luminous AuNPs is that these might not illustrate any surface plasmon due to the small number of free electrons and lack of genuine bands [314].

**Figure 11:**
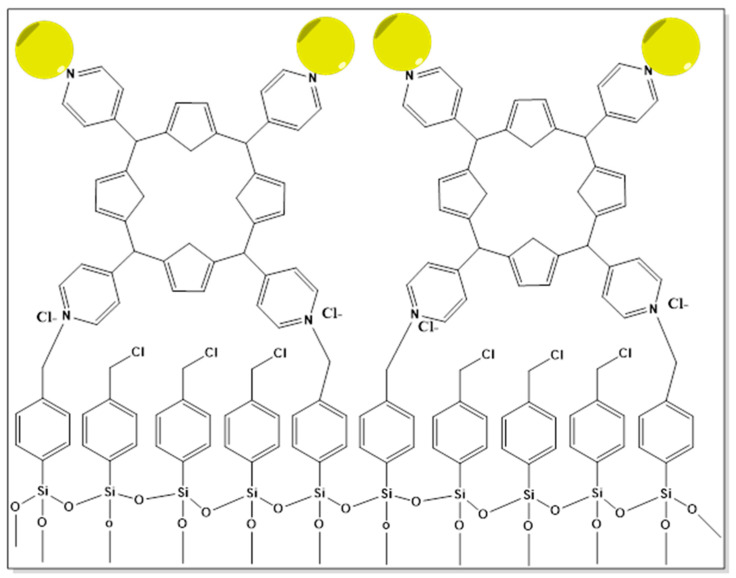
Illustration of Au@PH2TPP_SAM.

AuNPs can be built on surfaces, and the capping layer and cross-linker play a role in this process. The formation of a new composite assembly made up of an additional AuNPs monolayer conjugated with the porphyrin layer (Au@PH2TPP SAM) and a 5,10,15,20-tetra(4-pyridyl)-21H,23H-porphine monolayer (PH2TPP SAM) covalently anchored to silicon and silica functionalized substrates was demonstrated in a research study. According to their study, Au@PH2TPP_SAM, as shown in Figure 11, exhibits high surface plasmons caused by the AuNPs and luminescence signals caused by the porphyrin molecules [315].

AgNPs, sulfidation, and oxidation degradation effortlessly happen even at room temperatures. As a result, the LSPR band undergoes considerable spectral changes and/or broadening. Temperature, storage conditions, surface capping, light exposure, and solvent chemistry affect the degradation rate. Capping of AgNPs includes the use of agents such as citrate [316], polyvinylpyrrolidone (PVP) [317], benzyl dimethylammonium chloride(CTAC), methylammonium chromite (CTAB) [318], and thiolated poly(ethylene glycol)(PEG) [319]. AgNPs have been known to be protected from aggregation and dissolution by these capping agents. The lower protection fails when the nanoparticles are exposed to oxygen, sulfur, or other oxidizing species [320].

Storage and application of AgNPs in complex biological applications have been difficult, and they have been uncommonly used in cell culture assays. However, the unconstrained dissolution of AuNPs to Au+ ions and other soluble complexes is harmful to bacteria, fungi, and some cell types. As a consequence of their biological metabolism, living beings produce reactive oxygen species (ROS), for example, H2O2, singlet oxygen, superoxide anions, hydroperoxyl radicals, and sulfites (H2S, polysulfides), which readily trigger oxidation and sulfidation of AgNPs [321,322].

**Figure 12:**
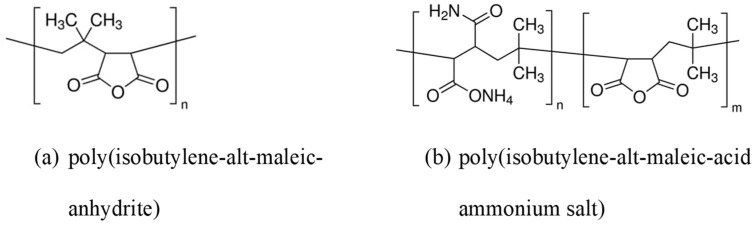
(**a**) Chemical structures of poly(isobutylene-alt-maleic-anhydrite) and (**b**) poly(isobutylene-alt-maleic-acid ammonium salt).

To curb this problem, protective layers are usually used, and metal oxides, biomolecules, and polymers are used to make these layers. Work has already been carried out on Ag@silica, Ag@titania, and a wide variety of Ag@polymer NPs based on Ag nanospheres [323]. A protein corona spontaneously forms when biofluids are exposed to the nanoparticle, which alters their cytotoxicity, biological activity, and cellular internalization. Proposed methods have been made to enhance the stability of Ag nanoparticles under different conditions, and one of them is the coating with other nanoparticles, including gold atoms [324]. A report by X. Zhuo et al. [325] on the synthesis of polymer-coated Ag nanorods of high colloidal and chemical stability was studied. They developed SERS nanotags employing dodecylamine-modified polyisobutylene-alt-maleic-anhydrite (PMA), in Figure 12, a protective layer for AgNPs that also permits the inclusion of a Raman reporter (RaR).

Due to their unique antibacterial and spectroscopic capabilities, silver nanoparticles (AgNPs) distinguish themselves from other metal nanoparticles. AgNPs’ chemical and physical properties might be tuned through organic covalent covering. Nicosia and colleagues, in their work, used a multi-step approach to coat silver nanoparticles [326]. 5,10,15-[p-(w -methoxy-polyethyleneoxy)phenyl] was used to functionalize the AgNPs. employing chloropropanethiol as a coupling agent to create 20-(p-hydroxyphenyl)-porphyrin (P(PEG350)3). They concluded that AgNP@P(PEG350)3 is a prospective multifunctional theranostic tool that combines the properties of AgNPs and P(PEG350)3. The nanosystem demonstrated its suitability as a portable pH sensor in aqueous solutions and its potential viability for applications in biological environments [213].

**Table 1:** Summary of the NP@porphyrin nanoconjugates potentially used for biological applications.

Porphyrin Derivative	Metal	Surface Modification of the NP	Potential Application	Effects of the Dual Theranostic Tool
5,10,15-p(ῳ-methoxypolyethyleneoxyphenyl)-20-p(hydroxyphenyl) porphyrin [326]	Ag	Poly(ethyleneglycol)methyl ether	Breast cancer	- Improved dispersibility in aqueous media, - High quantum yields Improved pH sensing
5,10,15,20-tetrakis(3-hydroxyphenyl)porphyrin [327]	Zn-Cu-In-S/ZnS QDs	No surface modification	Skin cancer	- Higher reduction in cell viability - Higher antibacterial effect
meso-tetra-(4-sulfonatophenyl) porphyrin [328]	Ternary copper indium sulfide/zinc sulfide (CuInS_2_/Zn) quantum dots	Polyethylene glycol	Prostate cancer	- Improved singlet oxygen generation and enhanced oxygen quantum yield
*meso*-tetrakis(4-hydroxyphenyl)porphyrin [329]	Superparamagnetic iron oxide NPs and Au	Polyethene glycol	Breast cancer	- High phototoxicity against
	Superparamagnetic iron oxide NPs and Au	Polyethene glycol	Breast cancer	- MCF-7 breast cancer cell lines
		Glutathione	Kidney cancer	- High cytotoxicity against high cytotoxicity against THP cells
5,10,15,20-tetrakis(3-hydroxyphenyl)porphyrin [327]	Zn-Cu-In-S/ZnS QDs			

### 6.2. Theranostic Applications of Modified Nanoparticles

Theranostic uses of customized nanoparticles promise a groundbreaking approach to personalized medicine, integrating therapeutic and diagnostic capabilities into a single platform. For example, gold nanoparticles functionalized with targeting ligands and therapeutic agents can work as imaging agents and drug delivery systems, allowing for precise tumor localization and tailored therapy [330]. Similarly, magnetic nanoparticles modified with specific antibodies can be utilized to detect cancer cells using magnetic resonance imaging (MRI) and destroy them using heat. Quantum dots, with their unique optical properties, can be designed to deliver drugs and examine cellular reactions in real time, providing data regarding treatment efficacy [331]. Furthermore, silver nanoparticles linked with fluorescent dyes and anticancer drugs can perform two functions, visualizing tumor areas and delivering cytotoxic chemicals directly to cancer cells, reducing side effects, and improving treatment outcomes [332]. These multifunctional nanoparticles have enormous potential for enhancing diagnostic accuracy, optimizing treatment approaches, and eventually expanding the area of precision medicine.

### 6.3. The Influence of Nanoparticles and Lipoproteins on Porphyrin Properties

Nanoparticles can significantly impact porphyrin properties, particularly their photophysical and photodynamic efficiency, making them more successful in applications such as PDT [333]. One significant influence is improved photophysical properties, such as enhanced stability and solubility of porphyrins when coupled with nanoparticles. When porphyrins are encapsulated in silica nanoparticles or coupled with gold nanoparticles, their absorption and fluorescence properties can be greatly improved [334]. These alterations can improve light absorption and fluorescence quantum yields, which are required for efficient PDT. Furthermore, nanoparticles’ surfaces can be functionalized to inhibit porphyrin aggregation, keeping their ability to create ROS effectively [335]. Moreover, using porphyrins with nanoparticles can improve photodynamic efficiency by enhancing photosensitizer targeting and distribution to tumor locations. For example, porphyrin-conjugated gold nanoparticles can use the increased EPR effect to accumulate more efficiently in tumor tissues [336]. This targeted administration guarantees that the photosensitizer is accumulated at the intended place, minimizing damage to surrounding healthy tissues. Nanoparticles’ distinctive features, such as magnetic or optical functions, can be used to enhance therapeutic efficacy. Magnetic nanoparticles, for example, can direct porphyrins to the tumor site when exposed to an external magnetic field [337].

Lipoproteins influence the distribution of porphyrins in the body, substantially impacting their efficacy in therapeutic applications such as PDT [338]. As hydrophobic molecules, porphyrins can bind to lipoproteins in the bloodstream, boosting their mobility and cellular uptake. High-density lipoproteins (HDL) and low-density lipoproteins (LDL) act as delivery agents, increasing the bioavailability and distribution of porphyrins to target tissues, including tumors [339]. The affinity of porphyrins for these lipoproteins can result in preferential uptake by cancer cells, which frequently overexpress lipoprotein receptors. This selective targeting amplifies the accumulation of porphyrins at the tumor site and reduces systemic toxicity by minimizing off-target effects [340]. In addition, the association with lipoproteins can stabilize porphyrins, limiting early degradation and in-creasing photodynamic efficiency. Understanding and utilizing the role of lipoproteins in porphyrin dispersion is crucial for optimizing PDT and other porphyrin-based therapies.

## 7. The Effect of the Structural Properties of the Metal Nanoparticles (Size, Shape, and Surface) on Their Biological Activity

Metal nanoparticles’ structural properties, such as shape, size, and surface characteristics, are critical to their cellular absorption and subsequent localization inside cellular compartments. These properties determine how nanoparticles interact with cell membranes, move through the intracellular environment, and eventually localize within certain cellular organelles such as the nucleus, cytoplasm, or mitochondria.

### 7.1. The Morphology of the Nanoparticles

The shape of metal nanoparticles influences their cellular uptake and intracellular dispersion. Spherical nanoparticles are often internalized more efficiently owing to their symmetric form, which allows for less difficult interaction with cell membrane receptors and endocytosis mechanisms [341]. Rod-shaped or elongated nanoparticles may have differing uptake dynamics, resulting in delayed internalization but potentially higher intracellular stability and retention [342]. The shape of nanoparticles might also influence the specific cellular compartments where nanoparticles localize; for example, rod-shaped nanoparticles may align with cytoskeletal structures, resulting in different intracellular trafficking pathways from spherical nanoparticles [343]. Furthermore, anisotropic shapes such as nanostars or nanocages can interact differently with biological components, perhaps improving interactions with specific organelles such as mitochondria or the nucleus, attributable to their distinct surface geometries and localized plasmonic fields [344].

### 7.2. The Size of Nanoparticles

Nanoparticle size is another important aspect that influences cellular absorption and intracellular distribution. Smaller nanoparticles (less than 10 nm) can easily permeate cell membranes and pass through nuclear pores, allowing direct contact with nuclear DNA and proteins [345]. This characteristic makes them attractive for gene therapy and targeted medication delivery to the nucleus. Larger nanoparticles (greater than 100 nm) can be internalized through phagocytosis, notably by macrophages, and used for targeted immune system delivery or cancer immunotherapy [346]. Nanoparticles of intermediate size (10–100 nm) frequently exhibit optimum cellular absorption via various endocytic pathways. They can efficiently escape endosomal entrapment, allowing for distribution in the cytoplasm and interaction with cytoplasmic components [347].

### 7.3. The Effect of the Surface Charge of the Nanoparticles on Biological Activity

The biological impact of the charge of metal nanoparticles, such as gold and silver nanoparticles, on cell membranes is extensive and complex. Positively charged nanoparticles often interact more strongly with negatively charged cell membranes, increasing cellular absorption while potentially disrupting membrane integrity [348]. This can cause increased permeability, oxidative stress, and the activation of cell death pathways. In contrast, negatively charged or neutral nanoparticles may have lower cellular absorption but can cause significant biological impacts via various pathways [349]. Understanding these interactions is critical because it reveals how nanoparticles might be tuned for specific therapeutic applications, such as targeted drug delivery or photodynamic treatment.

The positive charge on nanoparticles facilitates their interaction with the negatively charged phospholipid bilayer of cell membranes [350]. This strong electrostatic attraction enhances nanoparticle adsorption and penetration into cells. Once internalized, positively charged nanoparticles can disrupt the mitochondrial membrane potential, causing cytochrome c to be released into the cytosol [351]. This process is a key step in the intrinsic apoptosis pathway because it activates caspase-9, which then activates caspase-3, resulting in cell death [352]. Furthermore, disrupting membrane integrity can enhance intracellular ROS production, which promotes oxidative stress and death. Increased cell membrane permeability can also improve therapeutic agent delivery, increasing the efficacy of nanoparticle-based treatments. Moreover, positively charged nanoparticles can influence the expression of apoptosis-related oncogenes [353]. These nanoparticles can help transfer genetic material or drugs that regulate gene expression directly to the cell nucleus. As an example, they can upregulate pro-apoptotic genes like BCL-2-associated X protein (BAX) while downregulating anti-apoptotic genes like B-cell lymphoma 2 (BCL-2) [354]. This modulation is critical in tipping the balance towards cell death in cancer cells. Furthermore, positively charged silver nanoparticles have been shown to cause DNA damage and activate p53, a key tumor suppressor gene that controls the expression of multiple genes involved in apoptosis. Positively charged nanoparticles have the ability to cause apoptosis and decrease cancer cell proliferation by altering key signaling pathways and gene expressions [355].

Negatively charged silver or gold nanoparticles considerably influence cell membranes and apoptosis-related oncogenes in cancer cells, but in different ways than their positively charged counterparts. These nanoparticles have a decreased cellular absorption due to repulsion from the negatively charged cell membrane, potentially resulting in lower cytotoxicity. However, they can still cause apoptosis via different routes [348]. To activate the extrinsic pathway, negatively charged nanoparticles can engage with cell surface receptors and apoptosis signaling proteins, such as death receptors. This process involves the activation of caspase-8, activating downstream caspases such as caspase-3, resulting in cell death [356].

Furthermore, negatively charged nanoparticles can adsorb biomolecules, forming a protein corona that can influence cell signaling and gene expression [210]. This interaction can modulate apoptosis-related genes, such as upregulating pro-apoptotic genes and downregulating anti-apoptotic genes, thus increasing apoptosis in cancer cells. Furthermore, these nanoparticles can still produce ROS, adding to oxidative stress and mitochondrial malfunction, which are important factors in apoptosis [357]. Despite changes in absorption and primary interactions, negatively charged nanoparticles continue to be helpful in cancer therapy via altering apoptosis-related pathways and gene expression.

### 7.4. Coating of Metal Nanoparticles for Application in Biological Use

Coating methods are essential for reducing the toxicity of metal nanoparticles, increasing their biocompatibility, and improving their performance in biological applications. These tactics entail altering the surface of nanoparticles with various compounds to lower their intrinsic toxicity, prevent nonspecific interactions with biological components, and improve their stability and circulation in the body.

*Polyethylene Glycol Coating*: One of the most popular and effective coating techniques is to employ PEG, a hydrophilic polymer. PEGylation helps to form a steric barrier around the nanoparticles, minimizing protein adsorption and opsonization by the immune system, extending their circulation duration, and lowering immunogenicity [358]. PEG-coated nanoparticles have lower detection and uptake by the reticuloendothelial system (RES), reducing the toxicity associated with fast clearance and buildup in the liver and spleen [359]. Furthermore, PEGylation can augment the solubility and stability of nanoparticles in physiological conditions, reducing possible toxic effects.

*Biomolecule Functionalization*: Nanoparticles can be rendered more biocompatible and target-specific by coating them with biomolecules such as peptides, proteins, antibodies, or polysaccharides. For example, capping nanoparticles with albumin, a naturally occurring protein in the bloodstream, can disguise their surface and inhibit aggregation, lowering toxicity [360]. Antibodies or ligands can be added to nanoparticles to target certain cell types or receptors, guaranteeing that the nanoparticles interact with sick cells while avoiding healthy tissues [360]. This focused method improves therapeutic efficacy while minimizing off-target effects and related toxicity.

*Lipid-Based Coatings*: Another effective approach to minimize toxicity is encapsulating metal nanoparticles within lipid-based structures such as liposomes or lipid bilayers. Lipid coatings can mimic the natural makeup of cell membranes, making nanoparticles more biocompatible [361]. Liposomes can encapsulate nanoparticles and release them in a regulated manner, preventing fast disintegration and limiting interaction with non-target tissues. Additionally, lipid-coated nanoparticles can be made to fuse with cell membranes, allowing for their payload to be delivered directly into the cytoplasm, which is especially beneficial for drug delivery applications [362].

*Polymer Coatings*: Metal nanoparticles can be coated with various synthetic and natural polymers, including chitosan, dextran, and poly (lactic-co-glycolic acid) (PGLA). These polymers can amplify the stability, solubility, and biocompatibility of nanoparticles. Chitosan, for example, is a biodegradable polymer with low toxicity and intrinsic antibacterial characteristics, making it ideal for coating nanoparticles used in biomedical applications [363]. Another commonly used biodegradable polymer is PLGA, which enables the regulated and sustained release of therapeutic drugs, lowering dose frequency and systemic toxicity [364].

## 8. The Cancer Types That Are Responsive to Plasmonic Metal Nanoparticles

Metal nanoparticle efficiency varies greatly across cancer cell lines due to their distinct biological properties and genetic backgrounds. Several factors influence this selectivity, including receptor expression, cellular absorption methods, and metabolic pathways. The response of different cancer types to metal nanoparticles, notably gold and silver nanoparticles, is affected by several parameters, including metal type, cancer cell line selectivity, and the underlying biological pathways. Gold and silver nanoparticles have significant potential for treating breast, prostate, and lung cancers, whereas silver nanoparticles are beneficial against cervical, colon, and skin cancers [365]. Understanding the unique interactions between nanoparticles and cancer cells can help improve the design and application of nanoparticle-based medicines, increasing their efficacy and safety in cancer treatment. For example, in breast cancer treatment, AuNPs show great efficacy due to their large surface area, which allows for effective drug loading and targeted delivery [366]. AuNPs’ plasmonic characteristics improve photothermal treatment (PTT), in which localized heating caused by laser irradiation kills cancer cells. AuNPs can also be functionalized with targeting ligands, such as HER2 antibodies, to target HER2-positive breast cancer cells selectively, boosting treatment efficacy while minimizing damage to adjacent healthy tissues [367].

AuNPs are highly effective in prostate cancer therapy for delivering therapeutic drugs and PTT. Prostate-specific membrane antigen (PSMA)-targeted AuNPs improve therapeutic specificity and efficacy by precisely delivering AuNPs to prostate cancer cells while minimizing off-target effects [368]. Furthermore, studies have demonstrated that using AuNPs with radiotherapy improves treatment outcomes by increasing radiation-induced DNA damage in cancer cells. This combination therapy takes advantage of the radiosensitizing capabilities of AuNPs, making cancer cells more sensitive to radiation and increasing overall therapeutic outcomes [369].

Functionalizing AuNPs with peptides or antibodies specific to lung cancer biomarkers, such as epidermal growth factor receptor (EGFR), enables targeted therapy that lowers off-target effects while improving therapeutic outcomes [370]. This targeted delivery approach ensures that therapeutic drugs are focused on malignant tissue, increasing treatment efficacy while minimizing systemic side effects. Furthermore, the plasmonic features of AuNPs enable effective PTT, which adds to their promise for lung cancer treatment [371].

AgNPs are renowned for their antimicrobial properties, but they also have potent anticancer properties due to their ability to induce oxidative stress and death in cancer cells. AgNPs have demonstrated remarkable efficacy in cervical cancer by eliciting cytotoxicity by producing reactive oxygen species (ROS) and consequent death [372]. This process involves the impairment of mitochondrial activity in cancer cells, which causes cell death. The capacity of AgNPs to interfere with mitochondrial function is critical because it impairs cancer cells’ energy generation and survival systems, rendering them more vulnerable to apoptosis [373].

AgNPs show promise as a treatment for colon cancer because they induce ROS-mediated apoptosis and alter cellular homeostasis [374]. Their capacity to penetrate deep into tissues makes them ideal for targeting tumor cells in the colon. Similarly, they have shown efficacy against melanoma, a severe type of skin cancer. They cause cell death by oxidative stress and mitochondrial damage, amplifying the total cytotoxic effect [355]. Furthermore, AgNPs can synergize with traditional chemotherapies, lowering doses and minimizing side effects. This synergistic impact enhances treatment efficacy and reduces the risks associated with high-dose chemotherapy.

## 9. Combination Therapy

### 9.1. Chemotherapy and Photodynamic Therapy

Combining photodynamic therapy (PDT) with chemotherapy utilizing metal nanoparticles can greatly improve anticancer efficacy. Metal nanoparticles, such as gold nanoparticles (AuNPs), can be modified with photosensitizers and chemotherapeutic medicines. Photosensitizers produce reactive oxygen species (ROS) when exposed to light, which cause localized cell death. In contrast, chemotherapeutic medicines kill cancer cells via various methods, including DNA damage and cell division suppression [375]. This combination produces a synergistic effect in which PDT increases cancer cell permeability, allowing for deeper penetration and better efficacy of chemotherapeutic drugs. Furthermore, this method can reduce chemotherapy doses, lower side effects, and increase patient outcomes [376].

### 9.2. Photodynamic with Sonodynamic Therapy

PDT can be combined with sonodynamic therapy (SDT) to target cancer cells more efficiently. Ultrasound can activate metal nanoparticles functionalized with sonosensitizers to produce ROS, as light does in PDT [377]. This combination enables light and sound to create ROS, increasing oxidative stress within tumors. Furthermore, ultrasound may penetrate deeper into tissues than light, allowing for the treatment of larger, deeper-seated tumors. The synergistic action of PDT and SDT can result in greater tumor cell death and fewer side effects, as lower dosages of each therapy may be required [378].

### 9.3. Photodynamic with Immunotherapy and Radiotherapy

PDT with immunotherapy and radiotherapy can significantly enhance anticancer efficacy. Metal nanoparticles can be carriers for photosensitizers, immunomodulators, and radiosensitizers [379]. PDT can cause immunogenic cell death, releasing tumor antigens that activate the immune system. Immunotherapy can then boost this response by increasing the activity of immune cells fighting the tumor. Furthermore, radiotherapy can harm cancer cells and alter the tumor microenvironment, rendering it more vulnerable to immune attack. This multimodal approach capitalizes on the strengths of each therapy, ensuring a strong and long-lasting anticancer response while minimizing the side effects associated with high doses of individual treatments [380].

### 9.4. Photodynamic and Photothermal Therapy

PDT employs photosensitizers that produce ROS upon light activation, causing cell death predominantly through oxidative stress [381]. However, its efficacy may be reduced in hypoxic tumor regions with low oxygen. On the other hand, PTT uses metal nanoparticles to transform light into thermal energy, resulting in localized hyperthermia and the direct thermal ablation of cancer cells. When coupled, PDT can sensitize tumor cells to heat by altering cellular structures and increasing membrane permeability. PTT can improve blood flow in hypoxic zones, increasing ROS production during PDT. This complimentary action improves overall tumor-killing effectiveness and helps overcome each therapy’s limitations when used separately [382]. The combination of PDT and PTT in a single therapy regimen, frequently assisted by multifunctional nanoparticles, results in a synergistic strategy that maximizes tumor elimination while minimizing negative effects on surrounding healthy tissues.

PDT coupled with PTT employing metal nanoparticles is another effective anticancer method. Gold and other plasmonic nanoparticles are especially effective in this combination because of their high absorption of light and subsequent heat conversion. In this dual therapy, light activates the nanoparticles, generating ROS for PDT, followed by additional light exposure to cause hyperthermia for PTT [383]. The heat produced can directly ablate cancer cells and accelerate ROS-mediated damage, resulting in shrinkage of the tumor. This combination attacks cancer cells via numerous mechanisms, enhancing overall therapeutic efficacy while decreasing the probability of resistance [384].

## 10. Photothermal and Photodynamic Therapy Resistance

Resistance to PTT and PDT in cancer treatment can significantly limit their efficacy. In PTT, tumor cells frequently activate heat shock proteins (HSPs), aiding their survival under thermal stress. Furthermore, tumors can adapt by boosting blood flow to remove heat better, reducing the impact of localized hyperthermia [385]. Resistance to PDT is primarily due to the hypoxic nature of solid tumors, which reduces the oxygen required to form ROS. Furthermore, activating antioxidant defense mechanisms, such as increasing glutathione levels, can neutralize ROS and protect cancer cells from oxidative stress [382].

Alternatives to overcome PTT resistance involve employing HSP inhibitors that disable the cellular heat response, increasing thermal sensitivity. Combining PTT with anti-angiogenic medicines can limit blood supply to the tumor while maintaining higher local temperatures and promoting efficacy [335]. To combat resistance in PDT, techniques focus on increasing oxygen availability in the tumor microenvironment. This can be accomplished by using oxygen carriers, such as perfluorocarbon-based nanoparticles, or by administering treatments that normalize tumor vasculature to promote perfusion [386]. Furthermore, combining PDT with drugs that deplete antioxidants, such as glutathione inhibitors, can promote ROS formation and increase the treatment’s efficiency [387].

## 11. Conclusions and Future Perspectives

This paper reviews the use of gold and silver nanoparticles in photothermal therapy and the potential shortcomings in using either gold or silver nanoparticles for cancer treatment. This review thus highlights the need to functionalize them with different groups, such as polymers and organic ligands among others. The long-term biological behavior of noble metal nanoparticles in PTT is currently the main obstacle. MNPs nevertheless suffer from difficult biodegradation and probable toxicity despite having good photothermal efficacy, which can have serious side effects, such as, extended accumulation in organs. To effectively treat deep-seated cancers with PTT, it is necessary to overcome any physical restrictions to the depth of light penetration, often less than 1 cm beneath the skin. There is also a great possibility of integrating and combining different therapy methods. This review found that despite the potential of metal nanoparticles for hypoxia targeting, there are still major challenges that limit target efficiency, specificity to hypoxic tumor areas, reduction in off-target effects, biocompatibility, and safety for clinical translation. The development of biodegradable nanoparticles may improve the safety profile of these medicines. Furthermore, combining hypoxia-targeting MNPs alongside other therapeutic modalities, for example, chemotherapy, gene therapy, and immunotherapy, can synergistically impact treatment effectiveness. Combination of PTT with PDT provides a synergistic strategy to overcome the inherent resistance in each therapy. PTT can increase local oxygenation by increasing blood flow, which helps PDT generate ROS, whereas PDT can sensitize cells to heat to make them more vulnerable to PTT-induced damage. Development of multifunctional nanoparticles that can conduct PTT and PDT or sequentially activate each therapy shows potential to increase therapeutic efficacy and overcome resistance. These integrated techniques offer a complete strategy that can be used to address limitations and resistance associated with PTT and PDT for their use in cancer therapy. Future directions in pharmaceutical chemistry and nanomedicine could prioritize the incorporation of porphyrin derivatives, such as chlorin and expanded porphyrins, into advanced therapeutic modalities such as PTT, SDT, and PDT. The development of multifunctional nanoplatforms that encompasses these derivatives holds immense promise for specific, controlled, and efficient cancer treatment. It could challenge the inherent limitations of current clinical applications and open up new pathways for nanomedicine research. In this work, we showed that combination of photothermal and photodynamic therapy is possible, and more studies are necessary to determine the best way for optimal efficacy. Integration of porphyrins with either silver or gold nanoparticles will increase the biocompatibility, solubility, and cytotoxicity in nanoparticles. This will, in turn, have a synergistic effect and work better for therapeutic applications.
